# ACTIVE PARTICIPATION OF OLDER ADULTS AT POLITICAL RALLIES AS A SOURCE OF RESILIENCE: THE CASE OF THE ISRAELI PROTEST

**DOI:** 10.1093/geroni/igad104.3752

**Published:** 2023-12-21

**Authors:** Boaz Ben-David, Ortal Shimon-Raz, Yuval Palgi, lia Ring, Tchelet Bresslet

**Affiliations:** Reichman University, Herzliya, Tel Aviv, Israel; Reichman University, Herzliya, Tel Aviv, Israel; University of Haifa, Haifa, Hefa, Israel; Ashkelon Academic College, Ashkelon, HaDarom, Israel; Reichman University, Herzliya, Tel Aviv, Israel

## Abstract

The governing Israeli coalition suggested on Dec 2022 a reform plan that would fundamentally alter the system of checks and balances within Israeli society. According to law experts, the plan would effectively end liberal democracy in Israel. This turn of events sparked the largest protest movement in the 75year history of Israel. The current situation is unparalleled, and the mental health costs appear to be significant. However, the toll on older adults has not been directly examined yet. The breadth of the protest movement is remarkable. A July 2023 poll conducted by the Israel Democracy Institute estimated that almost a quarter of Israeli citizens had participated at a protest action at least once. It was primarily through participation at large rallies held weekly across the country - one of the main symbols of the protest movement. Interestingly, the survey reported a 37% participation rate among older adults, the highesy participation rate among all tested age groups. Since older Israelis are members of the founding generation of the state, their participation is not surprising. They feel threatened as their way of life and heritage are at risk. In the current study, we conducted a survey that examined mental health indices among older Israelis. Specifically, we wanted to test whether active participation at the protest could serve as a source for resilience in older age. Unfortunately, political turmoil is not unique to Israel. Thus, we hope that our findings could assist practitioners globally.

